# TANTIGEN 2.0: a knowledge base of tumor T cell antigens and epitopes

**DOI:** 10.1186/s12859-021-03962-7

**Published:** 2021-04-14

**Authors:** Guanglan Zhang, Lou Chitkushev, Lars Rønn Olsen, Derin B. Keskin, Vladimir Brusic

**Affiliations:** 1 Metropolitan College, Boston University, Boston, USA; 2 Department of Health Technology, Technical University of Denmark, Lyngby, Denmark; 3 Dana-Farber Cancer Institute, Harvard Medical School, Boston, USA; 4 School of Computer Science, University of Nottingham, Ningbo, China

**Keywords:** Neoepitope, Neoantigen, Tumor antigen, Cancer vaccine, Immunotherapy, T cell epitope prediction

## Abstract

We previously developed TANTIGEN, a comprehensive online database cataloging more than 1000 T cell epitopes and HLA ligands from 292 tumor antigens. In TANTIGEN 2.0, we significantly expanded coverage in both immune response targets (T cell epitopes and HLA ligands) and tumor antigens. It catalogs 4,296 antigen variants from 403 unique tumor antigens and more than 1500 T cell epitopes and HLA ligands. We also included neoantigens, a class of tumor antigens generated through mutations resulting in new amino acid sequences in tumor antigens. TANTIGEN 2.0 contains validated TCR sequences specific for cognate T cell epitopes and tumor antigen gene/mRNA/protein expression information in major human cancers extracted by Human Pathology Atlas. TANTIGEN 2.0 is a rich data resource for tumor antigens and their associated epitopes and neoepitopes. It hosts a set of tailored data analytics tools tightly integrated with the data to form meaningful analysis workflows. It is freely available at http://projects.met-hilab.org/tadb.

## Background

Advances in instrumentation and progress in immuno-oncology are driving a revolution in cancer care. New cancer treatment methods are emerging–targeted immunotherapies are among the most promising treatment options. Checkpoint blocking antibodies are currently providing stable cures to a subset of patients that could not be helped previously [1]. Chimeric antigen receptor (CAR) T cell/adoptive T cell therapies have been shown effective in some terminally ill patients [2]. Neoantigens are newly formed protein antigens that occur in individual patients, either from somatic mutations of genes or viral genes incorporated in the infected cell genome. These freshly emerged genes encode proteins that contain new T cell epitopes capable of inducing tumor-specific T cell recognition. Furthermore, it was demonstrated that the recognition of even a small number of epitopes by T cells might be sufficient to reject tumors in terminally ill patients through adoptive T cell therapy [3, 4].

T cells recognize antigens through T cell receptor (TCR), a surface protein composed of multiple peptide chains. A TCR has such high antigen specificity that it can recognize its cognate targets at a level of single amino acid difference. A highly diverse repertoire of TCR sequences ensures the effectiveness of the adaptive immune system [5, 6]. It was shown that T cell repertoire could serve as a biomarker of immune responses in cancer patients [7, 8]. TCR sequences with validated cognate antigens are essential for cell-based vaccine design. Vaccines that target neoantigens and personalized cancer immunotherapies are considered as the current clinical and research frontier in immuno-oncology [9, 10].

Tumor derived antigens that induce productive antitumor immune responses are known as tumor antigens (TAs) [11]. TAs can be divided into two main groups, tumor-specific antigens (TSAs) and tumor-associated antigens (TAAs). TSAs may be mutated tumor neoantigens [12], cancer-testis antigens [13], or oncofetal proteins [14]. TSAs are exclusively found in tumors and are not expressed in normal tissues. TAAs are normal proteins that are overexpressed in tumor cells as compared to the expression level in healthy cells [15]. Neoantigens are TAs that are no longer self. They are not tolerated by T cell immunity and are exclusively tumor specific. TAs have been extensively studied and offer high promise for cancer therapeutics design and serve as cancer diagnosis targets [9, 10, 16, 17]. The therapeutic landscape of cancer has recently been transformed by the emergence of effective immunotherapies [18, 19]. Despite these advances, any one form of immunotherapy studied and used to date was shown to benefit only a subset of patients. These immunotherapies facilitate T cell mediated immunity against the tumors. However, we lack an understanding of what defines the specificity of protective T cell immune responses they generate. Correct identification and cataloging of T cell antigens that aid tumor rejection will allow the development of highly effective personalized cancer vaccine immunotherapies and understand the protection mechanism. Current epitope prediction algorithms only forecast HLA processing and presentation but cannot predict antigenicity. They produce large numbers of positives that are biochemically active but functionally inert [20]. Because not all HLA presented epitopes are recognized by T cells, their inclusion in the vaccines and immunotherapies may reduce immunotherapy effectiveness. Validated data sets of T cell epitopes with the associated HLA restriction may also allow the development of improved epitope prediction algorithms [20, 21]. TCR sequences specific for TAs can be utilized to generate antitumor T cells for adoptive T cell therapy or as templates for building models of TCR-antigen recognition for neoantigens that show high similarity to known immune response targets.

To support the development of rationally designed epitope-based cancer vaccines, we previously developed TANTIGEN, a comprehensive web-based database cataloging more than 1000 T cell epitopes and HLA ligands from 292 different tumor T cell antigens [22]. In the current build, TANTIGEN 2.0 [23], we extended the coverage of immune response targets in the original set of TAs, added more than 100 new TAs, and a selection of their T cell epitopes and ligands. All T cell epitopes and neoepitopes included in TANTIGEN 2.0 have been experimentally validated.

## Implementation

### Data collection, annotation, and organization

We assembled TANTIGEN 2.0 by compiling the new data from the Cancer Antigenic Peptide Database [24] and recent publications reporting neoepitopes and neoantigens. Previously we included mRNA expression information of TAs by providing EST profiles from UniGene. However, NCBI retired the UniGene web pages in July 2019. Large scale open-access efforts, such as the Cancer Genome Atlas (TCGA) and the Human Protein Atlas (HPA), provided data for genome-wide expression analysis of individual genes in different tissues and cancers [25, 26]. The Human Pathology Atlas that was created as a part of the Human Protein Atlas program analyzed and cataloged expression profiles at both RNA and protein levels. Protein expression data are shown in normal tissue and major human cancers [27]. In TANTIGEN 2.0, we utilized data from the HPA to enrich available information on TAs. TCR sequences were collected from McPAS-TCR, a TCR sequence database associated with various pathologies and antigens based on published literature [28]. It contains more than 5,000 sequences of TCRs associated with multiple pathologic conditions, including cancer and their respective antigens in humans and mice. To ensure data integrity and avoid the proliferation of data errors from external databases, we manually checked the data against the original publications. For example, in McPAS-TCR, an HLA-A*0201 restricted T cell epitope YLEPGPVTA (IEDB ID: 74,638) was mistakenly described as A*01. The error was corrected when data were integrated into TANTIGEN 2.0.

### Bioinformatics tools

A set of bioinformatics tools has been integrated into TANTIGEN 2.0 to streamline data analysis. Users can look for antigens, epitopes, and HLA ligands using keyword searches. Sequence similarity searches can be performed using BLAST (Basic Local Alignment Search Tool) [29]. Sequence homology can be examined using multiple sequence alignment by MAFFT [30]. On-the-fly HLA binding prediction tools for 15 common HLA class I and class II alleles were integrated into TANTIGEN 2.0. They facilitate analysis of known immunogenicity in conjunction with predicted HLA binding and the prediction of additional potential epitopes. TANTIGEN 2.0 has a set of visualization tools that display the locations of peptides within their parent proteins. For each protein that contains point mutations, an interactive visualization tool shows a map of mutations in the tumor antigen sequences to provide a global view of all reported mutations for a given tumor antigen. For neoantigen entries, both the neoantigen fragment and the native sequence (called reference sequence) are included.

### Webserver

The TANTIGEN webserver interface was constructed using the KB-builder framework, which streamlines the development and deployment of web-accessible immunological databases [31]. TANTIGEN 2.0 serves as an integrator of relevant information critical for the design of highly personalized cancer vaccines and immunotherapies.

## Results

In TANTIGEN 2.0, we cataloged more than 1000 validated tumor T cell epitopes and over 500 HLA binding ligands (1,676 in total). The database hosts more than 4,000 antigen variants from 403 unique TAs reported in literature. Each record contains the information on tumor antigen sequence, variants (splice isoforms and mutation variants), known T-cell epitopes and HLA ligands, TCR information (if available), and literature references.

**Fig. 1:**
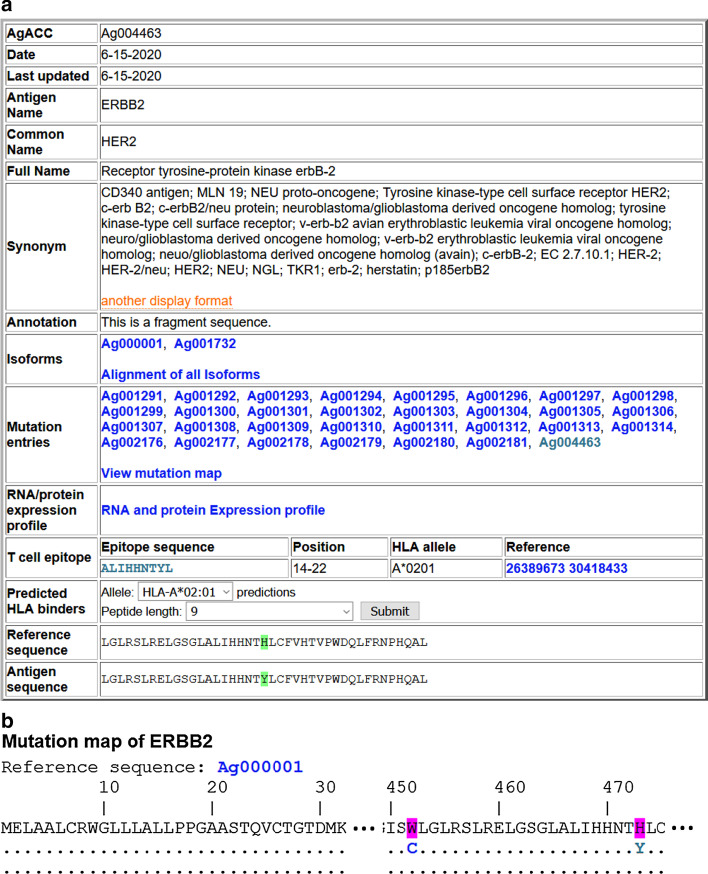

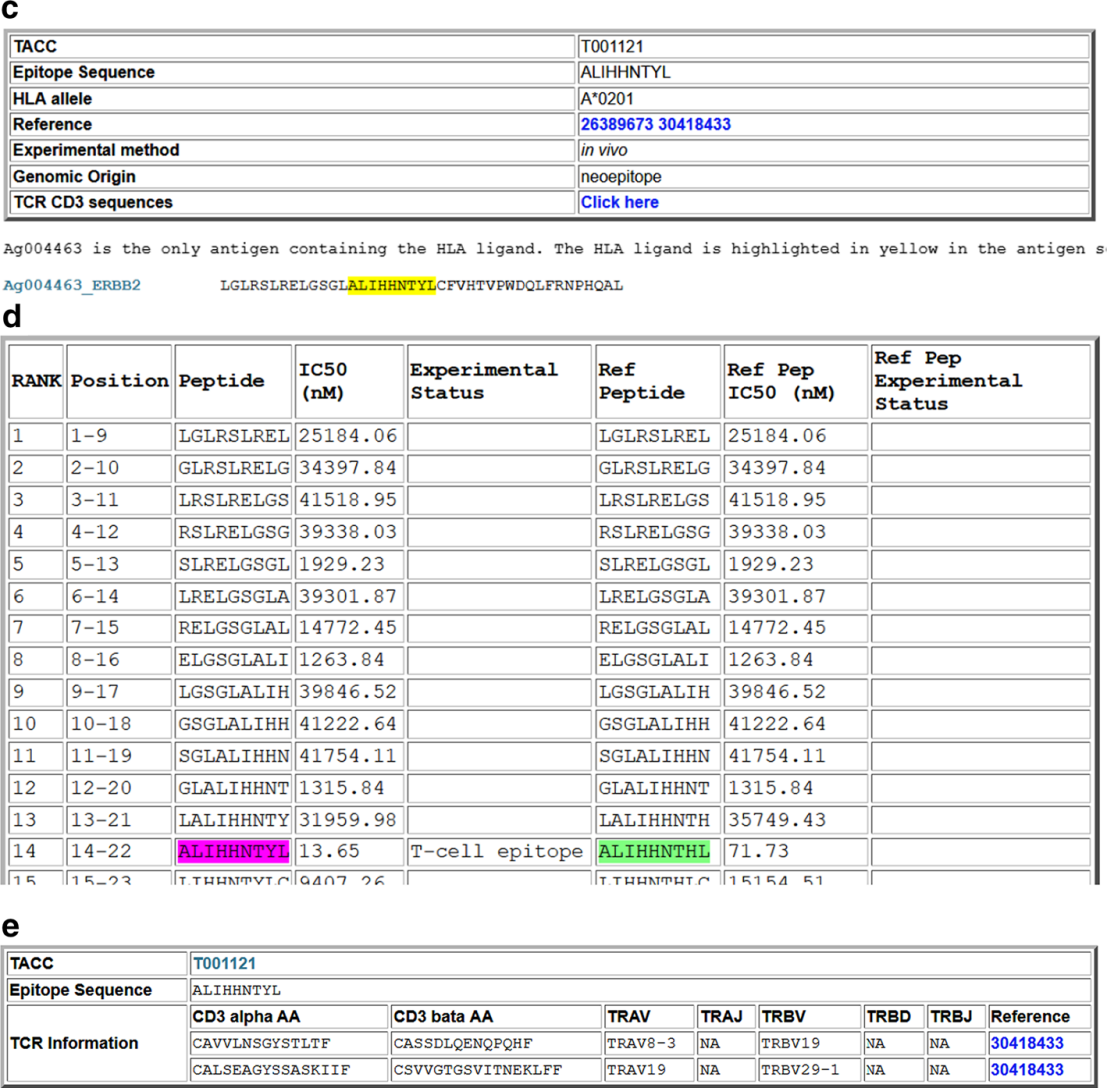
Example pages with neoantigen and neoepitope information: **a** HER2 neoantigen with a substitute mutation H473Y; **b** Part of the mutation Map; **c** HLA A*0201 restricted neoepitope; **d** On-the-fly HLA binding prediction with side-by-side comparison with the reference sequence. Predicted weak and strong binders are highlighted in green and magenta respectively; **e** TCR information

Around 50% of the antigens (201 out of 403) have substitution mutations, with about 25% (95) having more than one. The antigens have more than 100 substitution mutations include TP53 (1,311 mutations), CDKN2A (239), EGFR (169), CTNNB1 (111), TYR (111), and APC (103). The TA variants include 879 full-length sequences, 243 partial sequences, and 3,175 short fragments resulted from identified substitution mutations. Most defined short fragments are 41 aa long sequences with the mutation in the middle (position 21). Sometimes it is impossible to place the mutation position in the middle of the sequence. For example, in Ag000048, the mutation is at position 12 in the reference antigen. The length of these fragments is kept the same at 41. The reference sequence fragment is included for comparison with the mutated fragment; the mutation site is highlighted (Fig. 1a). The substitution mutation H473Y resulted in an A*0201 restricted neoepitope, as shown in Fig. 1b, c. The HLA binding prediction comparison in Fig. 1d shows that the peptide becomes a strong HLA-A*0201 binder instead of a weaker one due to the mutation. The TCR information for the neoepitope is shown in Fig. 1d.

## Conclusions

TANTIGEN 2.0 provides a rich data resource for tumor associated epitope and neoepitope discovery studies. It hosts more than 4,000 antigen variants from 403 unique TAs (a 38% increase comparing to TANTIGEN) and 1,676 T cell epitopes and HLA ligands (a 46% increase). TCR information for some T cell epitopes is included too. Integrated computational analysis tools in TANTIGEN 2.0 enable users to combine data and domain knowledge, use tailored bioinformatics tools, and simulate experiments. It represents a rich information resource for the study of cancer immunology and immunotherapy. All data and tools described here are available in TANTIGEN 2.0, an interactive open-access database (http://projects.met-hilab.org/tadb). The primary purpose of TANTIGEN2.0 is to support the design of neoantigen vaccine-based cancer immunotherapies. Immunological peptides from cancer-causing human viruses, such as Epstein-Barr virus (EBV), Human Papillomavirus (HPV), and Merkel Cell Polyomavirus (MCV), were not included in TANTIGEN. We developed EBVdb, HPVdb, and MCVdb to support studies on T cell immunology of EBV, HPV, and MCV [32–34].

## Availability and requirements

- Project name: TANTIGEN 2.0
- Project home page: http://projects.met-hilab.org/tadb
- Operating system(s): Platform independent.
- Programming language: Perl and PHP.
- Other requirements: None.
- License: Not applicable.
- Any restrictions to use by non-academics: None.

## Data Availability

All data are publicly available at http://projects.met-hilab.org/tadb. The organized datasets are available from the corresponding author on reasonable request.

## Abbreviations

BLAST: Basic local alignment search tool
CAR: Chimeric antigen receptor
EBV: Epstein-Barr virus
HLA: Human leukocyte antigen
HPA: Human protein atlas
HPV: Human papillomavirus
IEDB: Immune epitope database and analysis resource
MCV: Merkel cell polyomavirus
NCBI: National center for biotechnology information
TA: Tumore antigen
TAA: Tumor-associated antigen
TCGA: The cancer genome atlas
TCR: T cell receptor
TSA: Tumor-specific antigen
